# Effectiveness of martial arts exercise on anthropometric and body composition parameters of overweight and obese subjects: a systematic review and meta-analysis

**DOI:** 10.1186/s12889-020-09340-x

**Published:** 2020-08-17

**Authors:** Fabricio de Souza, Felipe Nunes Lanzendorf, Márcia Mendonça Marcos de Souza, Fabiana Schuelter-Trevisol, Daisson José Trevisol

**Affiliations:** 1 Postgraduate Program in Health Sciences, University of Southern Santa Catarina, Avenida José Acácio Moreira, 787, Bairro Dehon, Tubarão, Santa Catarina Caixa Postal 370 Brazil; 2 Degree in Biological Sciences, Leonardo da Vinci University Center, Capivari de Baixo, Santa Catarina Brazil; 3 Physical Education Degree, Leonardo da Vinci University Center, Capivari de Baixo, Santa Catarina Brazil; 4 Clinical Research Center, Hospital Nossa Senhora da Conceição, Tubarão, Santa Catarina Brazil

**Keywords:** Body mass index, Weight loss, Exercise, Physical activity

## Abstract

**Background:**

Obesity is considered a top public health concern, and its prevalence is growing every day. Thus, interventions to address this problem should be encouraged and further studied. In this regard, the aim of this review was to summarize the evidence of martial arts interventions to evaluate their effectiveness on the anthropometric and body composition parameters of overweight and obese subjects.

**Methods:**

A systematic literature search was conducted on January 26, 2020 using the PubMed, Medline, Lilacs, Cochrane, and Scielo databases. Reference lists of eligible articles and relevant reviews have also been examined. All randomized clinical trials on martial arts that evaluated the anthropometric and body composition parameters of overweight and obese subjects were included, and a narrative synthesis of eligible studies was conducted in accordance with PRISMA guidelines. The Downs & Black checklist was used to assess the quality of the studies. This review was registered in the International Prospective Register of Systematic Reviews (PROSPERO) (identifier CRD42018086116).

**Results:**

A total of 82 articles were identified from the initial search strategy. A further 2 articles were identified from the review of relevant bibliographies. Six studies encompassing 258 participants who were overweight or obese were included. Four studies reported Tai Chi practice, one study reported Kung Fu exercise, and another study reported martial arts exercise. The examined meta-analyses did not reveal significant benefits from martial arts practice over control groups after the experiment period for body mass index (− 1.34 kg/m^2^; 95% CI: − 2.72, 0.05), waist circumference (1.41 cm; 95% CI: − 0.72, 3.54) and percentage of body fat (− 0.75%; 95% CI: − 5.58, 4.08).

**Conclusion:**

The scarcity, heterogeneity, short intervention time, small sample size, and significant methodological limitations of the available studies do not allow to conclude whether martial arts are effective in the anthropometric and body composition parameters of overweight and obese individuals. This study highlights the need for more research to assess the benefits of martial arts for overweight and obese individuals.

## Background

Obesity is a chronic disorder of multifactorial origin, defined as an accumulation of abnormal or excessive fat that can be detrimental to health [1]. Currently, obesity is one of the most serious public health problems worldwide, being more prevalent than malnutrition itself [2]. According to the World Health Organization (WHO) [1], more than 1.9 billion adults were overweight, of whom more than 650 million were obese in 2016. These figures indicated that 39% of the adult world population was overweight, and 13% were obese [1]. In 2016, the WHO also estimated that 41 million children under the age of 5, and 340 million children and adolescents aged 5–19 years were overweight or obese worldwide [1].

Lifestyle interventions that provide a 5–10% reduction in body weight are recommended as a strategy to combat obesity [3, 4]. However, excess adipose tissue, especially visceral adipose tissue, poses greater risks to health than excess body weight [5]. Therefore, interventions aiming at weight loss should seek changes in anthropometric parameters and body composition, since the reduction of visceral adipose tissue decreases cardiovascular risks and metabolic issues [6].

Interventions for treating overweight and obese people include nutritional education and changes in lifestyle, which require increasing levels of physical activity and the reduction of sedentary behaviors [7]. The increase in energy expenditure through regular physical exercise is an interesting alternative for the treatment of overweight and obese people. Besides being a simple option to help control body weight [1], physical exercise can also produce positive health effects, such as cardiorespiratory fitness, reduction of inflammatory markers, elevation of anti-inflammatory markers, and improvements in cardiometabolic risk factors [8]. Studies have shown that aerobic exercise is the main form of exercise for obesity prevention and control [9–14]. However, some studies have reported important results from different treatment interventions for overweight and obese subjects, mainly interventions that used different intensity levels and the combination of aerobic exercises with strength exercises [15–18]. Thus, studies that investigate other forms of intervention with the use of physical exercise should be taken into account [15].

An organized activity that emerges as an alternative form of physical activity and that is gaining many supporters is the practice of martial arts [19–21]. Martial arts were developed for use on the battlefield [20, 21]. However, in the current context, they are used for a variety of purposes, including fitness, sports, self-defense, combat skills, meditation, character development, self-confidence, and health-related aspects [19–21]. In addition, they can be used as complementary or alternative therapy for some medical conditions [22]. Martial arts practitioners find great pleasure in performing physical activity, which is an extremely important factor, because in addition to encouraging participation, it contributes to better treatment results [23].

Despite the studies reporting and analyzing the benefits of practicing martial arts in the general population, scientific evidence regarding their use for the treatment of overweight and obese subjects is limited. Therefore, before recommending martial arts as a treatment for weight loss, it is important to analyze the scientific evidence for potential benefits and risks. For that reason, the purpose of this systematic review was to evaluate the effectiveness of martial arts exercise on the anthropometric and body composition parameters of overweight and obese subjects.

## Methods

The PRISMA Statement (Preferred Reporting Items for Systematic reviews and Meta-Analyses) [24] was used to conduct and report the systematic review. This review was registered in the International Prospective Register of Systematic Reviews (PROSPERO) (identifier CRD42018086116). However, no study protocol was published in a journal before.

### Eligibility criteria

The inclusion criteria were the following: (1) randomized controlled trials study design; (2) published in English, Spanish or Portuguese; (3) martial arts exercise, isolated or combined with any other practices; (4) overweight or obese participants (body mass index [BMI] ≥ 85 percentile for children and adolescents, and BMI > 25 for adults); (5) use of a quantitative method to determine at least one of the relevant measurements of anthropometric and body composition parameters (BMI, body-fat percentage, or waist circumference).

### Search strategy

To ensure the inclusion of relevant literature, the electronic search was conducted in different databases, namely PubMed, Medline, Lilacs, Cochrane, and Scielo in January 26, 2020. The adopted search strategy included a combination of the following Medical Subject Headings (MeSH) terms: obesity AND martial arts. Keyword iterations related to these terms were also used. Example of the search strategy in PubMed: Obesity [all fields]; Obes* [all fields]; Overweight [all fields]; #1 OR #2 OR #3; Martial arts [all fields]; Martial Fitness [all fields]; Martial exerc* [all fields]; #5 OR #6 OR #7; #4 AND #8 (supplementary file 1). Also, the reference lists of the included studies were examined as an additional check for potential studies that could be used in this review.

### Selection of studies and data extraction

Initially, all titles and abstracts were screened independently by two researchers (F.S. and F.N.L). Each researcher selected the studies that met the inclusion criteria. Full texts were read by 2 authors (FS, FNL) and the final list of eligible studies was compiled. If discrepancies between the 2 researchers occurred those items were discussed with a third researcher (DJT) to achieve a consensus. After selecting the articles, the following data were extracted: author, date of publication, language, sample size, demographic characteristics (Country/setting, age and ethnicity), BMI, body composition, intervention and control, and outcomes. The researchers were not blinded to the journal titles and paper authors for the data extraction process.

### Bias risk assessment

The methodological quality of the studies was evaluated independently by two researchers (FS and FNL), using the Downs & Black checklist [25]. This assessment tool has 27 scoring items to assess the strength of reporting, external validity, internal validity, and statistical power of randomized and non-randomized studies of health care interventions (Additional file 2). In that tool, the maximum score that can be received is 32. The score obtained by each study was divided by 32 and multiplied by 100 to provide a ‘Study Quality Percentage’, considering the following breakdown: high quality (66.7% or more); medium quality (between 50.0–66.6%), and low quality (less than 50.0%) [26]. Doubts and divergences between the researchers were resolved through consensus and mediated by a third researcher (DJT).

### Data analysis

The systematic review data were analyzed and presented employing a narrative synthesis approach. We examined the year of publication, country of origin, study design, sample characteristics, types of martial arts selected for intervention, outcome measures and treatments applied to the control groups. The overall effect sizes for each outcome were determined using meta-analytic approaches if at least 2 studies assessed the specific outcome. The Review Manager 5 software (Version 5.4, The Nordic Cochrane Centre Copenhagen) was used, and random effects models were applied. Meta-analyses were conducted to examine the effects of martial arts compared with control groups. Mean differences (MD) with 95% confidence intervals (CI) were reported. Attempts were made to obtain missing data from the studies’ authors by email.

Statistical heterogeneity between the studies was determined using I^2^ statistics, and the magnitude of heterogeneity was categorized as I^2^ = 0 to 24%: low; I^2^ = 25 to 49%: moderate; I^2^ = 50 to 74%: substantial; and I^2^ = 75 to 100%: considerable heterogeneity [27, 28]; and the x^2^ test was used to assess the statistical significance of heterogeneity between trials. In the face of the low power of this test in small samples, a *P* value ≤0.10 was regarded to indicate significant heterogeneity [29].

## Results

**Fig. 1:**
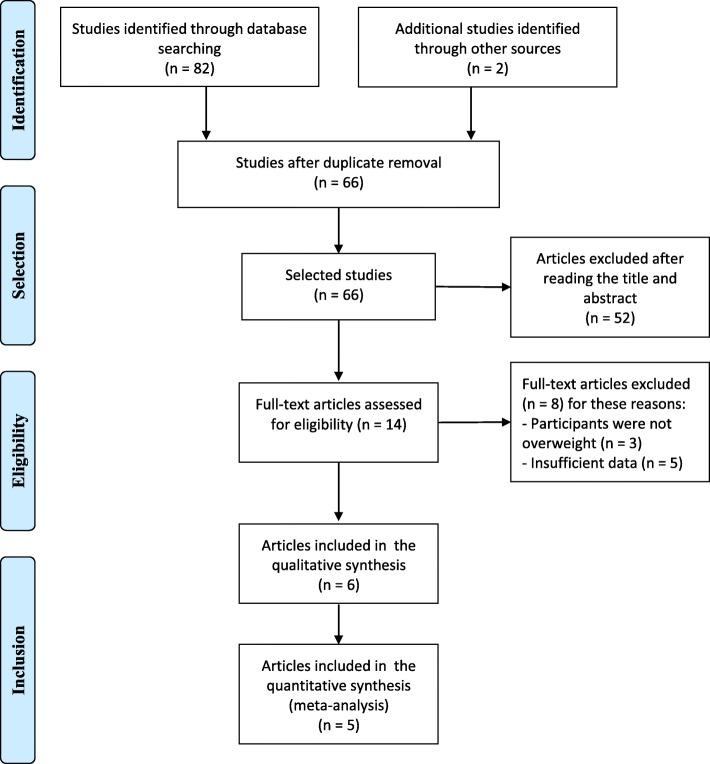
PRISMA – Study selection process. Body mass index. Body-fat percentage. Waist circumference

The electronic search initially retrieved 82 titles. Two additional articles were found in the reference section of the review articles. After duplicate articles were removed, 66 articles were screened by reading the titles and abstracts. Of them, 52 articles were excluded because they did not meet the inclusion criteria. The remaining 14 articles were selected for further review. After full reading, 8 studies were excluded (3 did not address overweight or obese subjects and 5 did not evaluate body composition). The study selection process is shown in the PRISMA [24] flow diagram (Fig. 1). A total of 6 studies were included in this systematic review (Table 1).

**Table 1:** Characteristics of the intervention studies that included martial arts

Study	Quality	Country/ Setting	Body composition	BMI	Sample n (total/F); Mean age, years (SD or range); ethnicity	Intervention	Control	Main results	D
Tsang et al. [30] 2009	21 medium	Australia/Hospital	BMI/DEXA	≥85%	20/12; 13.1 (2.1); NR	Kung Fu 3 x week - 1 h	Tai Chi 3 x week - 1 h	Difference between groups: waist circumference, with a lower circumference in the CG.	24
Beebe et al. [31] 2013	11 low	USA/University	BMI	≥30–49.9 kg/m^2^	26/26; 61.5 (6.0); NR	Tai Chi 3 x week 45 min + NE 1 x week 45 min	NE 1 x week 45 min	Difference within groups: both groups showed a significant decrease in body-weight, and waist circumference. The CG had a significant decrease in the BMI and hip circumference.	16
Chen et al. [32] 2010	18 medium	China/Hospital	BMI	≥30–35 kg/m^2^	117/54; 58.2 (6.0); Asian	Tai Chi 3 x week - 1 h	Conventional exercises 3 x week - 1 h	Difference between groups: BMI, with a decrease in the IG. Difference within groups: the IG showed a significant decrease in the BMI.	12
Chyu et al. [33] 2013	20 medium	USA/Community	BMI/bioimpedance	≥25 kg/m^2^	47/47; 40.6 (6.2); NR	MAE 3 x week - 1 h	Daily activities	Difference between groups: FFM and MM, with a lower amount in the IG.	12
Dechamps et al. [34] 2009	21 medium	France/Hospital	BMI/BOD POD	≥30 kg/m^2^	21/21; 44.4 (11.9); NR	Tai Chi 1 x week - 2 h + diet + NI + PI + MI	Conventional exercises 1 x week - 2 h + diet + NI + PI + MI	Difference between groups: The IG showed a decrease in fat (kg) and percent body fat.	10
Katkowski et al. [35] 2013	15 low	USA/Laboratory	BMI/DEXA	≥30–49.9 kg/m^2^	27/27; 61.5 (5.9); NR	Tai Chi 3 x week 45 min + NI 1 x week 45 min + diet	NI 1 x week 45 min + diet	Difference within groups: weight, BMI, fat (kg), percent body fat, and waist circumference, with a decrease in both groups.	16

*DEXA* Dual-energy X-ray absorptiometry, *BM* behavior modification, *BMI* body mass index, *ER* energy restriction, *F* female, *PE* physical education, *NE* nutritional education, *NI* nutritional intervention, *PI* psychological intervention, *MI* medical intervention, *NR* not reported, *RT* resistance training, *SD* standard deviation, *CI* confidence interval, *ST* strength training, *D* Duration in weeks, *MAE* martial arts exercises, *BOD POD* air displacement, *IG* intervention group, *CG* control group, *FFM* fat-free mass, *MM* muscle mass

### Characteristics of the studies

Six randomized clinical trials (*n* = 6) [30–35] were included in this systematic review (Table 1). The selected studies were published between 2009 and 2013. Three of them were conducted in the United States (50%) [31, 33, 35], one in Australia (16.66%) [30], one in France (16.66%) [34], and one in China (16.66%) [32]. The analysis encompassed 258 overweight or obese subjects. Sample sizes ranged from 20 to 117, with a mean of 43 (SD = 37.55). The majority of the participants were women 72.5% (*n* = 187), with a mean age of 46.5 years (SD = 18.66). BMI was taken as a criterion in all studies for identifying the overweight or obese sample 100% (*n* = 6), but different criteria were adopted in various studies to define overweight and obesity. Most studies (66.66%) defined a BMI ≥ 30 (*n* = 4) as a criterion for inclusion, one study (16.66%) defined a BMI ≥ 25, and one study (16.66%) defined a BMI ≥ 85th percentile.

### Interventions

Regarding the type of intervention, four studies (66.66%) reported Tai Chi practice as an intervention technique [31, 32, 34, 35], one study (16.66%) reported Kung Fu practice [30] and another study (16.66%) reported different martial arts styles [33]. Number of intervention sessions per week ranged from 1 to 3, with a mean of 2.66 (SD = 0.82). The duration in weeks ranged from 10 to 24, with a mean of 15 (SD = 5.02). It should be mentioned that no study used the same intervention methodology, differing between them in relation to the proposed interventions, frequency and duration of the sessions, additional interventions, and treatments in the control groups.

Tai Chi was the most common martial arts training applied as intervention in the treatment group 66.66% (*n* = 4). The Yang style 24-posture Form was the most widely studied 75% (*n* = 3) [31, 34, 35], and only one study by Chen and colleagues [32] reported the Chen style 99-posture form Tai Chi as the sole intervention in the treatment group. Three studies (75%) [31, 34, 35] reported additional interventions besides Tai Chi training in the treatment group. Beebe and colleagues [31] adopted nutrition education as an additional intervention technique. Dechamps and colleagues [34] developed psychological and medical interventions, besides nutritional education, and diet. Katkowski and colleagues [35] employed nutritional interventions composed of lectures and diets.

Different interventions were performed in the control groups applying Tai Chi as the main intervention in the treatment group [31, 32, 34, 35]. Beebe and colleagues [31] used nutrition education as intervention in the control group. Two studies (50%) [32, 34] reported conventional training as intervention in the control group, but the study by Dechamps and collaborators [34] also adopted nutritional, psychological, and medical intervention, in addition to conventional training. Katkowski and colleagues [35] performed nutritional interventions composed of lectures and diets.

Two studies (33.33%) [30, 33] developed other forms of martial arts. Tsang and colleagues [30] investigated the effects the Kung Fu Choy Lee Fut Hung Sing Gwoon style as intervention and the Tai Chi Yang style 24-posture form as placebo for the control group. Chyu and colleagues [33] studied martial arts exercise composed of traditional techniques of different martial arts styles as intervention in their study and recommended that the participants in the control group should maintain their daily activities.

#### Participation

Three studies have not reported the participation rate in their interventions [31, 32, 34]. Tsang and colleagues [30] reported that the participation rate in the interventions was less than 50%. Chyu and colleagues [33] reported participation rates of 83.4%. Katkowski and colleagues [35] subdivided the participation rate because of their multiple interventions. They reported that the participation rate of the martial arts intervention group was 73.1% for the Tai Chi technique and 78.1% for the dietary intervention. The participation rate in the control group was 86.5% for the dietary intervention.

#### Adverse events

Two studies [31, 35], which accounted for 33.33% of the studies included in this systematic review, did not report adverse events in their interventions. Dechamps and colleagues [34] reported the absence of adverse events in their study. Chen and colleagues [32] reported there were very few adverse events or lesions. Chyu and colleagues [33] reported the occurrence of muscular pain, knee pain, and shoulder pain in the first 2 weeks of intervention. Tsang and colleagues [30] reported two falls that occurred during the intervention period, noting that wearing inappropriate clothes was the cause of one of the falls.

### Quality of studies

A quality index was assigned to each of the studies included in this systematic review. The score ranged from 11 to 21, with a mean of 17.66 (SD = 3.98) [25], as shown in Table 2 and Additional file 2. Quality rating indicated that 66.66% of the studies were rated medium quality (*n* = 4), and 33.33% were rated low quality (*n* = 2); none was rated high quality. Assessment of study quality in this systematic review revealed the following negative factors: no identification of the main confounding factors, lack of treatment control and details of adverse events, poor analysis of the participants lost in the follow-up, absence of random sampling to represent the entire population from where the participants were recruited, no blinding for those responsible for assessing the outcomes, lack of adequate adjustment for the confounding variables in data analysis from which the findings were drawn, and inadequate sample size.

**Table 2:** Study quality assessment using the Downs & Black tool

Studies Item	Tsang et al. [30]	Beebe et al. [31]	Chen et al. [32]	Chyu et al. [33]	Dechamps et al. [34]	Katkowski et al. [35]
D&B: 1	1	1	1	1	1	1
D&B: 2	1	1	1	1	1	1
D&B: 3	1	1	1	1	1	1
D&B: 4	1	1	1	1	1	1
D&B: 5	0	0	0	0	0	0
D&B: 6	1	1	1	1	1	1
D&B: 7	1	1	1	1	1	1
D&B: 8	1	0	1	1	1	0
D&B: 9	1	0	0	1	1	0
D&B: 10	1	1	1	1	1	1
D&B: 11	0	0	0	0	0	0
D&B: 12	0	0	0	0	0	0
D&B: 13	1	0	0	0	0	0
D&B: 14	1	0	0	0	1	0
D&B: 15	1	0	0	1	1	0
D&B: 16	1	1	1	1	1	1
D&B: 17	1	0	1	1	1	0
D&B: 18	1	1	1	1	1	1
D&B: 19	0	0	0	1	0	1
D&B: 20	1	1	1	1	1	1
D&B: 21	1	0	1	1	1	1
D&B: 22	1	0	1	1	1	1
D&B: 23	1	1	1	1	1	1
D&B: 24	1	0	0	1	1	0
D&B: 25	0	0	0	0	1	0
D&B: 26	1	0	0	1	1	0
D&B: 27	0	0	3	0	0	1
**Total**	**21**	**11**	**18**	**20**	**21**	**15**

*D&B* Downs & Black item;

### Effectiveness of martial arts practice

Effectiveness assessment of martial arts practice in overweight and obese subjects are presented as follows.

#### BMI

All studies of this systematic review assessed BMI (100%), but only Chen and colleagues (16.66%) [32] reported a significant difference between the intervention groups (31.3 kg/m^2^; SD = 4.2) and control groups (32.8 kg/m^2^; SD = 4.4) after the intervention period, with a lower BMI in the intervention group. However, four studies (66.66%) [31, 32, 34, 35] reported a significant difference between the ‘before and after’ intervention period (Tables 3, 4).

**Table 3:** Anthropometric and body composition parameters of the intervention groups that took martial arts. Values expressed as mean (±SD)

Reference	Baseline								Follow-up							
	N	BW	BMI	WC	HC	WHR	FM (%)	FM (kg)	N	BW	BMI	WC	HC	WHR	FM (%)	FM (kg)
Tsang et al. [30]	12	84.9	32.1 (6.7)	99.1 (17.9)	NR	NR	44.6 (6.6)	37.5 (15.0)	11	87.2	32.7 (7.8)	100.2 (19.3)	NR	NR	43.1 (7.6)	37.4 (15.7)
Beebe et al. [31]	16	88.6 (13.95)	33.7 (4.8)	105.6 (9.1)	121.04 (11.9)	NR	NR	NR	13	86.3 (14.5)^a^	32.8 (5.0)	102.0 (10.4)^a^	118.7 (11.6)	NR	NR	NR
Chen et al. [32]	56	NR	33.5 (4.8)	NR	NR	NR	NR	NR	50	NR	31.3 (4.2)^a^	NR	NR	NR	NR	NR
Chyu et al. [33]	23	96.1 (15.2)	36.14 (5.42)	NR	NR	NR	NR	45.06 (10.68)	16	96.1 (17.1)	35.74 (5.97)	NR	NR	NR	NR	44.05 (11.69)
Dechamps et al. [34]	11	95.9 (14.7)	37.4 (4.8)	NR	NR	NR	48.5 (4.3)	46.6 (9)	11	92.5 (15.9)	36.0 (5.6)	NR	NR	NR	45.6 (5.1)^a^	42.6 (9.5)^a^
Katkowski et al. [35]	14	90.0 (3.9)	34.3 (5.1)	106.8 (2.6)	NR	NR	34.9 (1.2)	43.0 (2.5)	14	87.7 (4.0)^a^	NR^a^	103.6 (3.0)^a^	NR	NR	30.9 (1.4)^a^	41.0 (2.6)^a^

^a^ *p* < 0.05; *N* participants, *BW* body weight, *BMI* body mass index, *WC* waist circumference, *HC* hip circumference, *WHR* waist-to-hip ratio, *FM* fat mass, *NR* not reported, *SD* standard deviation

**Table 4:** Comparison of anthropometric and body composition parameters between treatment and control groups. Values expressed as mean (SD)

Study	Group	BMI		Waist circumference		% fat mass	
		Before	After	Before	After	Before	After
Tsang et al. [30] 2009	Treatment	32.1 (6.7)	32.7 (7.8)	99.1 (17.9)	100.2 (19.3)	44.6 (6.6)	43.1 (7.6)
	Control	34.0 (7.0)	34.2 (7.2)	106.5 (16.7)	103.6 (17.1) ^a^	47.7 (5.3)	47.4 (5.6)
Beebe et al. [31] 2013	Treatment	33.7 (4.8)	32.8 (5.0)	105.6 (9.1)	102.0 (10.4) ^b^	NR	NR
	Control	34.8 (2.9)	33.4 (3.1) ^b^	107.1 (9.0)	101.9 (10.6) ^b^	NR	NR
Chen et al. [32] 2010	Treatment	33.5 (4.8)	31.3 (4.2) ^a, b^	NR	NR	NR	NR
	Control	33.2 (4.1)	32.8 (4.4)	NR	NR	NR	NR
Chyu et al. [33] 2013	Treatment	36.1 (5.4)	35.7 (5.9)	NR	NR	NR	NR
	Control	35.6 (7.1)	36.2 (7.7)	NR	NR	NR	NR
Dechamps et al. [34] 2009	Treatment	37.4 (4.8)	36.0 (5.6) ^b^	NR	NR	48.5 (4.3)	45.6 (5.1) ^b^
	Control	38.5 (7.3)	39.3 (8.2)	NR	NR	50.3 (7.0)	48.1 (4.7)
Katkowski et al. [35] 2013	Treatment	34.3 (5.1)	NR^b^	106.8 (2.6)	103.6 (3.0) ^b^	34.9 (1.2)	30.9 (1.4) ^b^
	Control	34.8 (2.9)	NR	107.1(2.5)	102.0 (2.9) ^b^	33.5 (1.8)	28.1 (1.1) ^a, b^

^a^Significant difference between treatment and control groups; ^b^Significant difference between pre and post intervention; *p* < 0.05; *BMI* body mass index, *NR* not reported, *SD* standard deviation

**Fig. 2:**
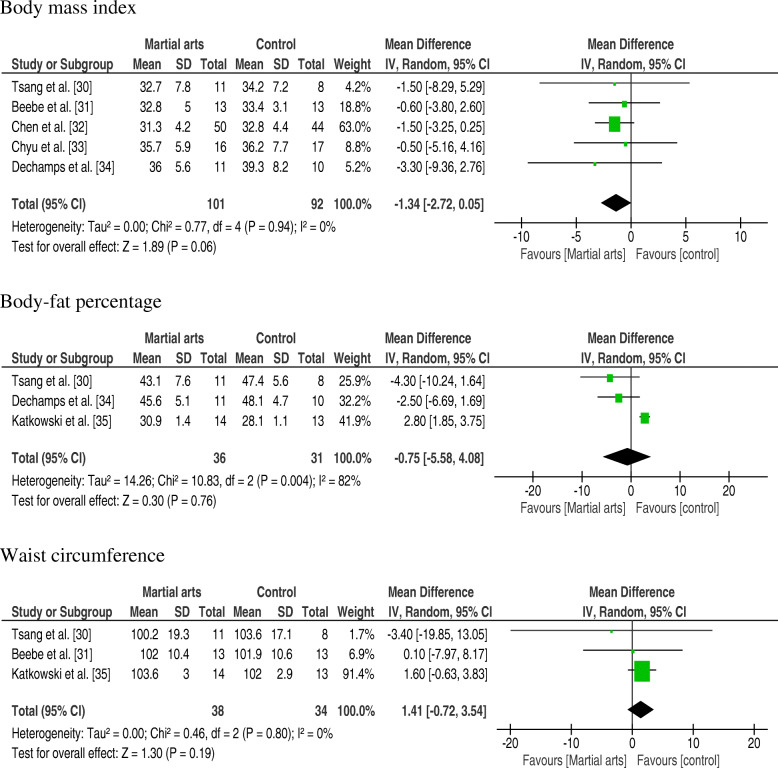
Forest plot and effect sizes for martial arts compared with controls for body mass index, body-fat percentage and Waist circumference

Five studies (83.33%) [30–34] in the meta-analysis assessed the outcome of martial arts interventions in relation to BMI (Fig. 2). The meta-analysis indicated a trend to reduce mean BMI for the groups that received martial arts interventions − 1.34 kg/m2 (95% CI: − 2.72, 0.05; I^2^ = 0%), with *p* = 0.06 and low level of heterogeneity.

#### Body-fat percentage

Three studies (50%) assessed the percentage of body fat [30, 34, 35] (Tables 3, 4). However, only Dechamps and colleagues (16.66%) [34] reported a significant difference between the intervention groups (45.6%; SD = 5.1) and control groups (48.1%; SD = 4.7) after the intervention period, with a lower percentage of body fat in the intervention group (*p* < 0.005). Nonetheless, two studies (33.33%) [34, 35] reported a significant difference between the ‘before and after’ intervention period.

Three studies (50%) [30, 34, 35] assessed the outcome of martial arts interventions in relation to body-fat percentage (Fig. 2). The examined meta-analysis did not reveal significant benefits from martial arts practice over control groups after the experiment period for percentage of body fat − 0.75% (95% CI: − 5.58, 4.08; I^2^ = 82%), with *p* = 0.76 and high level of heterogeneity.

#### Circumferences

Three studies (50%) assessed waist circumference [30, 31, 35] (Tables 3, 4). However, only Tsang and colleagues (16.66%) [30] reported a significant difference between the intervention groups (100.2 cm; SD = 19.3) and control groups (103.6 cm; SD = 17.1) after the intervention period, revealing a waist circumference increase in the intervention group (before 99.1 cm ± 17.9 and after 100.2 cm ± 19.3) and a decrease in the control group (before 106.5 cm ± 16.7 and after 103.6 cm ± 17.1). Two studies (33.33%) [31, 35] reported a significant difference between the ‘before and after’ intervention period for both martial arts intervention and control groups.

Three studies (50%) [30, 31, 35] assessed the outcomes of martial arts interventions in relation to waist circumference (Fig. 2). The meta-analysis showed no statistically significant difference in waist circumference between the martial arts intervention and control groups (1.41 cm; 95% CI: − 0.72, 3.54; I^2^ = 0%), with *p* = 0.19 and low level of heterogeneity.

## Discussion

This systematic review evaluated the efficacy of martial arts training on the anthropometric and body composition parameters of overweight and obese subjects. It can be inferred that martial arts are a promising form of intervention for the treatment of overweight and obese subjects, since the meta-analysis showed a tendency to decrease BMI in those groups that received martial arts interventions (Fig. 2). However, there were no significant results from martial arts practice over control groups after the experiment period for waist circumference and percentage of body fat (Fig. 2). It should be pointed out, though, that most of the control groups received some intervention as well. Although mixed results have been reported, these findings were similar to those observed in other studies that analyzed the effects of aerobic exercise in postmenopausal overweight and obese women [36]. No systematic reviews were found assessing the effects of martial arts on the anthropometric and body composition parameters of overweight and obese subjects. To our knowledge, this is the first study to investigate the topic. However, there is a review that investigated the effectiveness of martial arts for metabolic diseases [37] and another that investigated the Efficacy of Tai Chi and qigong for the prevention of stroke and stroke risk factors [38].

The results observed in this systematic review regarding the anthropometric and body composition parameters were incongruent, since some studies verified significant differences after the intervention period, whereas others did not. One factor that may have influenced the results was related to the age of the participants, since most of the studies [31–35] were conducted on older adults and elderly people. Only one study focused on adolescents [30], with several methodological problems. Another factor that might have influenced the results was the fact that most studies investigated solely Tai Chi style [31, 32, 34, 35]. Given that Tai Chi is a low-intensity physical exercise providing low-energy metabolism, with low energy-yielding metabolism, it does not favor beneficial changes in body composition [39].

A recent systematic review has investigated the benefits provided by the practice of hard martial arts in adults, and found they were safe and could provide several benefits, even for the elderly population [21]. However, these benefits could not be confirmed in the present review, as only two studies [30, 33] reported different martial arts interventions, and both had several methodological limitations. The study by Tsang and colleagues [30] was the only one included in this review that focused on adolescents as research subjects. The authors used Kung Fu as martial art intervention and Tai Chi as a placebo for the control group. That study had the longest duration (24 weeks), but the outcomes regarding changes in body composition and anthropometric parameters were not those expected, since the only difference between the intervention and control groups was the decrease in waist circumference in the control group that had Tai Chi intervention. This fact can be attributed to several factors related to the study, such as unintentional changes in the diet, number of participants (smaller than the required number by the sample size calculation – β error), low participation rate in interventions (< 50%), and low intervention intensity due to the configuration of the training sessions [30].

Another study by Chyu and colleagues [33] adopted a differentiated intervention. The authors used various martial arts exercises as an intervention technique for women aged 30 years or older in the premenopausal period. This study also did not find positive outcomes in relation to changes in body composition, a fact that can be attributed to the short intervention period (12 weeks), lack of energy intake control, or the exercise intensity during the interventions, which were planned to be maintained between 50 and 70% of maximum heart rate [33]. This level of exercise intensity only allows modest changes in body composition, especially if administered alone. Thus, this was an inadequate treatment method for overweight or obese people [40].

The studies showing positive outcomes regarding changes in body composition used Tai Chi intervention followed by nutrition education and dietary intervention [34, 35]. This was corroborated by Thorogood and colleagues [40] who found that moderate-intensity exercises accompanied by dietary and nutrition interventions can provide changes in body composition and be used as treatment for overweight and obese subjects [40]. However, Chen and colleagues [32] employed only martial arts as an intervention technique and found a significant reduction in BMI. Nonetheless, that study enrolled a sample composed of people aged between 40 and 70 years, BMI of ≥30–35 with type 2 diabetes mellitus. This specific population can respond differently to stimuli, since physical exercises are considered an important protection for people suffering from this disease [41]. Najafipour and colleagues [42] stated that regular aerobic exercise could improve body composition for people with type 2 diabetes mellitus, and Tai Chi could be considered a low-intensity aerobic exercise. In this population, regular exercises also provide a significant benefit in insulin sensitivity, which may persist beyond 72 h after the last exercise session [43].

### Limitations of this systematic review

One of the challenges encountered in this review was the fact that all the studies adopted different methods, with different frequency and duration of training sessions, treatment lengths of time, and additional treatment interventions in the control groups, making it difficult to compare the studies. Another difficulty in pursuing the objectives of this review was the fact that most of the studies focused on Tai Chi as the martial art intervention, which is a low-intensity martial art.

This systematic review did not identify any major adverse events in any of the studies that composed it. However, 33.33% of the studies did not mention whether there were adverse events in their interventions, which could lead to misinterpretation of the safety of the interventions.

The results of the quality assessment of the studies that composed this review show that most studies obtained a medium-quality rating and none of them obtained a high score, which indicates more rigorous research studies are needed to determine the efficacy of the interventions (Table 2). Two of the review studies did not provide information about the sample size calculation [31, 34]. Two studies used sample sizes smaller than those required for the study [30, 33]. None of the reviewed studies employed a randomized recruitment, and one of the studies did not report how the recruitment method was conducted [31].

Given that this was the first systematic review to examine the effects of martial arts interventions on overweight and obese subjects, all randomized clinical trials published up to January 2020 that met the inclusion criteria were encompassed in the review, regardless of their quality assessment.

### Implications for research

Despite the limitations in this study, it should be highlighted that this was the first systematic review to assess martial arts exercise interventions for overweight and obese subjects.

This systematic review presented important outcomes from the studies that composed it. However, although the interventions have been relatively safe, it is important to conduct further research involving other types of martial arts, with more rigorous study designs, involving nutritional intervention with control of energy intake, assessment and control of daily activities, detailed description of the interventions and adverse events, larger sample sizes, and different research populations. These measures would allow us to obtain more conclusive results on the effects of martial arts as a treatment method for overweight and obese subjects. They would also help obtain results that can be generalized with confidence.

## Conclusion

The scarcity, heterogeneity and significant methodological limitations of the available studies do not allow to conclude whether martial arts are effective in the anthropometric and body composition parameters of overweight and obese individuals. Martial arts can be an important tool for the treatment of overweight and obese people, as the results of this systematic review indicate that martial arts are probably safe and may offer benefits related to anthropometric and body composition parameters. However, the results are limited and should be used with caution. Because of this, further research is needed before we can generalize the findings regarding the efficacy of martial arts for maintaining healthy anthropometric parameters among overweight and obese subjects.

## Supplementary information

**Additional file 1.** Search strategy.

**Additional file 2.** Checklist for measuring study quality (Downs & Black).

## Data Availability

Not applicable.

## Abbreviations

BMI: Body mass index
MeSH: Medical Subject Headings
PRISMA: Preferred Reporting Items for Systematic reviews and Meta-Analyses
PROSPERO: International Prospective Register of Systematic Reviews
WHO: World Health Organization
