# Supplementary material for: Effectiveness of martial arts exercise on anthropometric and body composition parameters of overweight and obese subjects: a systematic review and meta-analysis
Source: BMC Public Health. 2020 Aug 17;20:1246. doi: 10.1186/s12889-020-09340-x (PMC7433112; doi:10.1186/s12889-020-09340-x)
Supplement: Supplementary file 1 — Additional file 1. Search strategy. [file 12889_2020_9340_MOESM1_ESM.docx]

**Supplementary file 1.** Search strategy

**PubMed**

1. Obesity [all fields]

2. Obes* [all fields]

3. Overweight [all fields]

4. #1 OR #2 OR #3

5. Martial arts [all fields]

6. Martial Fitness [all fields]

7. Martial exerc* [all fields]

8. #5 OR #6 OR #7

9. #4 AND #8
